# Guy's Hospital Appeal

**Published:** 1905-04-15

**Authors:** 


### GUY'S HOSPITAL APPEAL.

### SPECIAL COURT OF GOVERNORS—£33,000 SUBSCRIBED.

Lord Revelstoke presided at a Special Court of Governors of Guy's Hospital on Friday, the 7th inst., to which donors to the special appeal now before the public were invited. There was a large attendance.

Letter from the Prince of Wales.

Having opened the proceedings, the chairman called upon the treasurer, Mr. Cosmo Bonsor, to read the following letter received after a "surprise visit" paid by H.R.H. the Prince of Wales:—

Marlborough House, Pall Mall, S.W.:
March 31, 1905.

Dear Bonsor,—The Prince of Wales desires me to let you know how much pleased he was with his visit to Guy's Hospital on Monday last.

Though, as you know, the visit of the President was entirely a surprise one, there was nothing His Royal Highness saw to which he could take the slightest exception, and he considers such a state of things reflects great credit on all concerned.

His Royal Highness trusts that the fact of this efficient working and good administration of Guy's Hospital will beneficially affect the appeal for increased funds which the Hospital has recently made to the public.

Believe me, yours very truly,
Arthur Bigge.

Letters were read also from the Prime Minister, Lord Aldenham, formerly president of the hospital, Dr. Frederick Taylor, Mr. Alfred Beit, the Bishop of Southwark, the Hon. Walter Rothschild, Mr. Julius Werner, and others, all expressing deep sympathy with the object of the meeting.

The Chairman briefly explained how the meeting came to be called. He said the question naturally arose as to the most appropriate manner in which thanks might be expressed to the donors whose names appeared on the first list of subscriptions. The Governors had always felt that the importance of the work performed by Guy's Hospital, and the necessity of augmenting its endowments by support from the general public, had only to be made known to secure their prompt recognition. For this reason it was felt that no more fitting course could be adopted than to hold a public meeting close to the spot where the distinguished founder lay at rest amid the strenuous life and energy of his great charity, and, while signifying their gratitude to those who had so promptly responded, to appeal to the general public to follow their example and aid in carrying on the work.

The aim was to complete the labour of the past ten years and to restore Guy's Hospital to that sound financial position in which it was placed by its distinguished founder. He did all that lay within his power, both by wealth and intelligence, and for generations the benefits of his charity had been freely bestowed; benefits which had been shared, not alone by those "poor persons" whom Thomas Guy had nearest to his heart, but indeed by every class of society. It was to the high efficiency of Guy's Hospital and the constantly increasing demands upon it that the need for additional support beyond its original endowments was due; and there was no hospital in London more essential to the well-being of its great population than that on whose behalf he had the honour to appeal.

For its due preservation and full activity, a capital sum of £100,000 was needed, and an increase in the ordinary income of at least £15,000 per annum. The former amount was required to provide the balance of the cost of indispensable improvements, and also to give effect to the humane desire of his Majesty the King that all the wards in this hospital should be open for the reception of the sick poor.

The Governors were most grateful to H.R.H. the Prince of Wales for the tribute he had paid to the efficient working and good administration of Guy's Hospital, a tribute which had been confirmed by the Council of King Edward's Hospital Fund for London, by all expert critics, and by the Press. He was very glad to have the opportunity of thanking them for their powerful aid, and of expressing appreciation of the sympathy and approval which the efforts of the board had met with in every quarter.

The first list of donations which the Treasurer would announce would, he was confident, considering the short time they had been at work, be regarded as exceedingly generous. They had, indeed, every reason for encouragement and satisfaction, but he would add the reminder that this was the beginning, not the end, of the matter. There must be no relaxation of effort till the last farthing of the needful amount appealed for was forthcoming, and this might mean a task which could not but be long and arduous, unless, indeed, some generous benefactor should be moved to an act of munificence which would entitle him to rank with the illustrious founder in his claim on the gratitude of his fellow men.

Lord Rothschild then moved the following resolution:—

That this meeting of Governors and supporters of Guy's Hospital commends the present appeal to the immediate and practical recognition of the whole community throughout London and the provinces.

Lord Rothschild said, his pleasure in supporting the appeal was enhanced by the fact that an opportunity was afforded him of expressing his admiration of the long roll of illustrious men educated in the Medical School, who, by the learning and ability there acquired, and their devotion to their patients, had brought health and happiness to untold numbers outside the hospital. Guy's was in the same case as many old charities, in that, while the income derived from rent and investments had fallen off, the demands on space and surgical and medical efficiency had enormously increased; the appeal therefore, had his warmest support.

Mr. A. C. Coles, in seconding the resolution, paid a high tribute to the efficiency of the hospital, which he attributed in large measure to the wise administrative capacity of Sir Cooper Perry, the courage of the treasurer, and the efficiency of the medical, surgical, and nursing staff. Referring to the leading article in The Hospital for April 1st, Mr. Coles said it was the opinion of the writer, an expert in hospital matters, that Guy's was a hospital worthy of being copied, and this should be borne in mind by intending donors to the appeal.

Mr. Frank Debenham, Dr. Hale White, and the Hon. Sydney Holland having warmly supported the resolution, it was carried unanimously.

The Chairman having called upon Mr. Cosmo Bonsor to announce the first list of subscribers,

The Treasurer said he would like to refer to one or two matters which had not as yet been dealt with, and which demanded mention. He wished to acknowledge and make known the sympathy and unanimous support which the efforts of the Governors had received from the whole of the medical and surgical staff of the hospital. Not only was this the case, but the staff made a large annual contribution to the funds of the hospital, an act of generosity on their part which he regretted was not brought out in evidence before Sir Edward Fry's committee on the financial relations between the London hospitals and their medical schools. And help from its own people was not limited to the staff—old Guy's men, present students, and the nursing staff were all rendering spontaneous and ready and welcome help. With the valuable aid of the *Guy's Hospital Gazette* they had enabled the board to start an ambitious scheme in the form of a Million Shilling Fund, and the students, some 600 in number, acting on their own initiative, had pledged themselves to an organised canvass of South London. Surely there could not be better testimony than this to the deserving nature of their case. With reference to the Million Shilling Fund, £50,000 raised in this way must mean a host of contributions. Guy's own people were giving it a splendid start, either themselves or through their friends, but the board would shortly have to commend the fund to general support. Their first method had been to distribute remittance cards and envelopes in which contributions of one or more shillings might be forwarded direct to the hospital. Then large lists had been prepared for collections in offices and business houses. These he hoped would be much in demand, and he was happy to state that the Bank of England had set the example by asking for 50. Large donations were naturally desired, but the many small ones would be needed too, and what he wanted to point out was, that through the Million Shilling Fund the board had provided a way in which almost everyone might take a useful and honourable part in the great work of restoring financial prosperity to Guy's Hospital. The treasurer then announced the first list of contributions, amounting to a grand total of £33,002 18s.

A vote of thanks to the Chairman, proposed by General Sir Richard Harrison, G.C.B., and seconded by the senior surgeon, Mr. R. Clements Lucas, brought the proceedings to a close.

